# Electromagnetic microwave generation by acoustic vibrations gives rise to nanoradiophotonics

**DOI:** 10.1038/s41598-021-87389-3

**Published:** 2021-04-08

**Authors:** M. A. Shevchenko, M. A. Karpov, A. D. Kudryavtseva, D. V. Rozinskii, N. V. Tcherniega, S. F. Umanskaya

**Affiliations:** 1 P.N. Lebedev Physical Institute of the RAS, Leninskii pr., 53, Moscow, 119991 Russia; 2 Steklomash JSC, ul. Moiseenko, 11, Orekhovo-Zuevo, Moscow Region, 142600 Russia

**Keywords:** Photoacoustics, Nonlinear optics

## Abstract

The development of new methods for generating pulsed electromagnetic microwave radiation is currently an actively developing area of research. Schemes for microwave radiation generation with optical pumping are of great interest. In this paper we propose and experimentally demonstrate principally new method for photonic generation of microwave electromagnetic radiation. This method is based on the use of radiation of charged submicron particles oscillating at their own acoustic frequency. Laser radiation of the optical range implements an effective buildup of acoustic vibrations of submicron particles forming the system under study, according to the Raman mechanism.

## Introduction

One of the most actual problems of modern radio-photonics is the development and creation of systems that make it possible to interface optical circuits with microwave devices^1,2^. Such conjugation allows to expand significantly the spectrum of functional capabilities of microwave systems operating in the gigahertz frequency range. Great interest in such systems, as a rule, is associated with the possibility of their application for radar tasks. But their applications have been also actively developing in several other areas, both purely practical, such as the creation of wireless and satellite networks, signal processing and visualization systems, and in tasks related to the solving fundamental physical problems in astrophysics, precision spectroscopy and optomechanics^3–5^. For this purpose a lot of methods for effective microwave generation are being developed. Despite the large number of existing classical microwave generators such as the klystron, traveling wave tubes, gyrotrons, backward wave oscillators and others^6^, development of new types of devices allowing the generation of microwave radiation is a very urgent task today. The focus is on improving the spectral, energy and temporal characteristics of radiation, as well as reducing the cost of the devices themselves. A special place among them is occupied by methods based on using a dual wavelength laser source with two wavelengths separated by a microwave frequency^7,8^.

The aim of this work was to demonstrate experimentally the possibility of experimental implementation of the generation of pulsed electromagnetic radiation in the microwave range in a system of submicron particles with optical pumping. The main idea, experimentally realized in this work, is to use natural acoustic vibrations of nanoscale and submicron particles with a charge or polarized in an external field as a source of microwave electromagnetic radiation with frequency coinciding with acoustic one. The spectrum of natural acoustic frequencies of submicron particles lies in the gigahertz range, and of nanoscale particles in the terahertz range. To calculate the eigenvalues of the acoustic frequencies in the case of submicron particles, we use the approach proposed in the works of Lamb^9^ and currently actively used for the analysis of low-frequency Raman scattering (LFRS)^10,11^. For the case of liquid droplets of nano and submicron size, the so-called “droplet model” is used, which gives frequency values somewhat lower than the Lamb model. For experimental verification of the applicability of a model, as a rule, the method of LFRS of light is used, which makes it possible to obtain the values of the natural frequencies of acoustic vibrations considering the matrix in which the particles are located. A stimulated analogue of the LFRS process—stimulated low-frequency Raman scattering (SLFRS)^12,13^ of light can also be used to obtain information on the values of acoustic frequencies. SLFRS can also be used to generate acoustic vibrations of the studied system of nano or submicron particles. Note that in the case of SLFRS, the system of nano or submicron particles performs coherent acoustic vibrations, and their phasing is carried out through the re-emission field.

### Samples

In this work synthetic opal matrices were used as the active medium. Opal matrix is a material with spatial modulation of both optical and acoustic properties on a scale of several hundred nanometers. It has photonic band gaps in the visible spectral range for modes propagating in certain directions. The total photonic band gap with a zero density of photonic states can be realized in inverted opal matrices with a refractive index contrast greater than 2.85. Nevertheless, opal matrices are convenient to use for studies of nonlinear interaction of light radiation with matter due to the possibility to determine easily the position of band gaps and control their properties. The voids between quartz spheres in opal matrices are octahedral and tetrahedral in shape. By filling these cavities with liquids with different refractive indices, one can control the parameters of the photonic band gap (its position in the spectrum and contrast) and increase the efficiency of nonlinear processes due to a change in the density of photon states near the edge of the band gap^14,15^.

Three-dimensional opal matrices were grown by the method of slow crystallization of a monodisperse colloid solution of α-SiO_2_ globules synthesized by the modified Stöber method^16^. The resulting sediment after drying in air was annealed at 125 °C for 1 h and then at 750 °C for 2 h. The sample of the opal matrix had dimensions of 5 × 5 × 4 mm. The (111) plane of the face centered cubic lattice (FCC) of the opal matrices corresponded to the surface of the natural growth of the photonic crystal and was characterized by the absence of visible defects at a scale comparable with the spot of laser radiation on the surface of the sample (in the experiments on microwave generation laser beam diameter in the focal plane was about 5 µm). Synthetic opal matrices consist of close-packed amorphous silicon dioxide spherical particles with a characteristic size of 200 to 400 nm. We used opal matrices with the diameter of silicon oxide globules close to 270 nm. The standard deviation of the size distribution of globules was about 5%.

**Figure 1:**
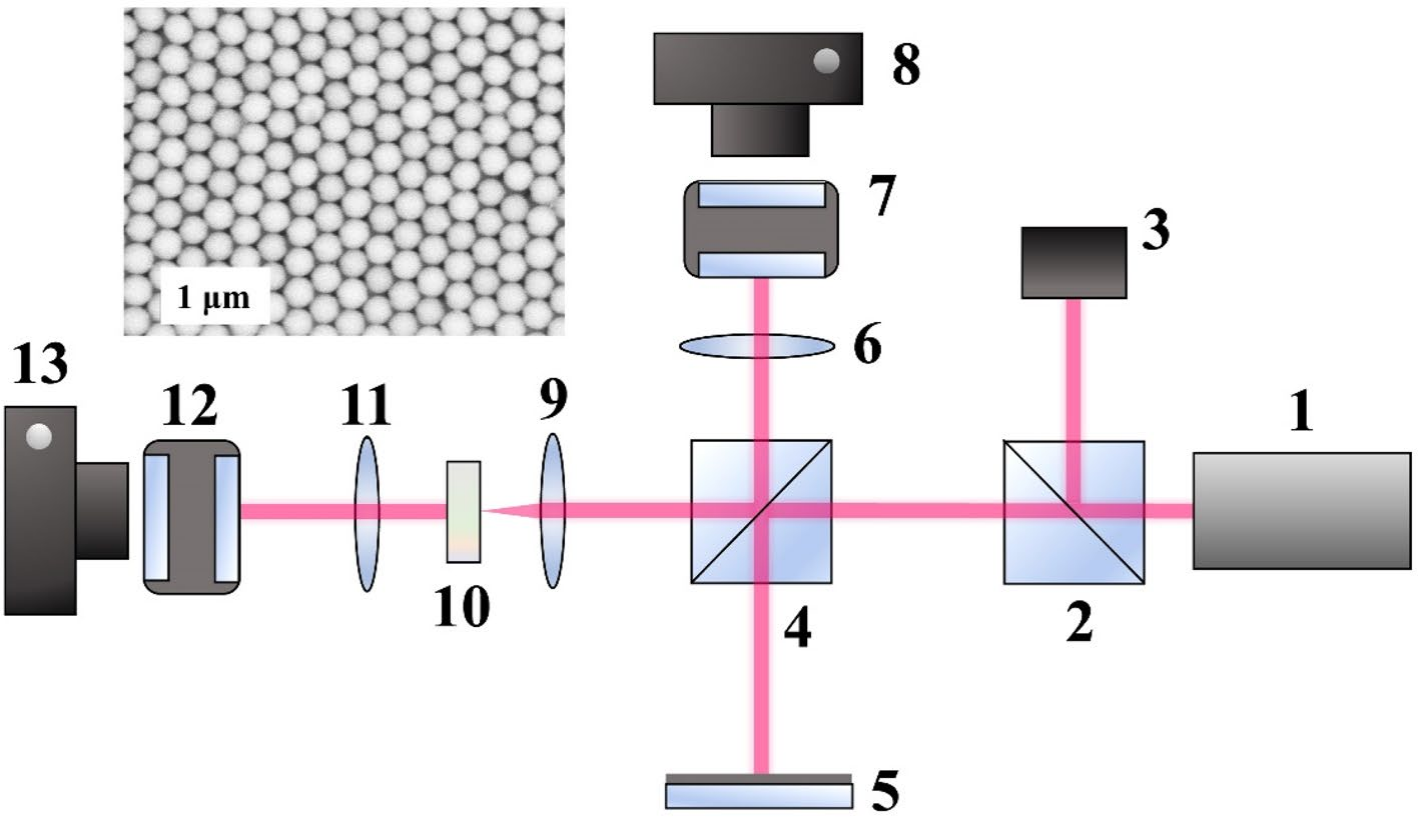
Experimental setup. 1—laser; 2, 4—transparent glass beam splitters with transmission to reflection rate 92:8; 3—the system for electromagnetic radiation parameters control; 5—mirror; 6, 9, 11—focusing systems; 7, 12—Fabry–Perot interferometers; 8, 13—digital cameras; 10—sample. Inset—SEM image of the opal matrix surface.

SEM image of the surface of the opal matrix used is shown in the inset in Fig. 1.

### Methods of SLFRS study and results

SLFRS in opal matrices can be used to determine the values of the frequencies of the natural acoustic vibrations of quartz globules. Under the influence of powerful laser pulses in the visible range, coherent excitation of acoustic vibrations in the system is possible with frequencies lying in the gigahertz range and manifesting themselves in the SLFRS spectra because of modulation of optical radiation by a low-frequency component. For SLFRS excitation ruby laser (λ = 694.3 nm, τ = 20 ns, E_max_ = 0.3 J, Δν = 0.015 cm^−1^, divergence 3.5 × 10^–4^ rad) was used. An experimental setup for determining the frequencies of natural acoustic vibrations of synthetic opal matrices is shown in Fig. 1.

SLFRS spectra were registered by Fabry–Perot interferometers with different base (distance between mirrors) which gave possibility to change the range of dispersion from 5 to 30 GHz. Thus, we could register both lines with small frequency shift (0.5 ÷ 2 GHz) and larger frequency shift (10 ÷ 20 GHz).

**Figure 2:**
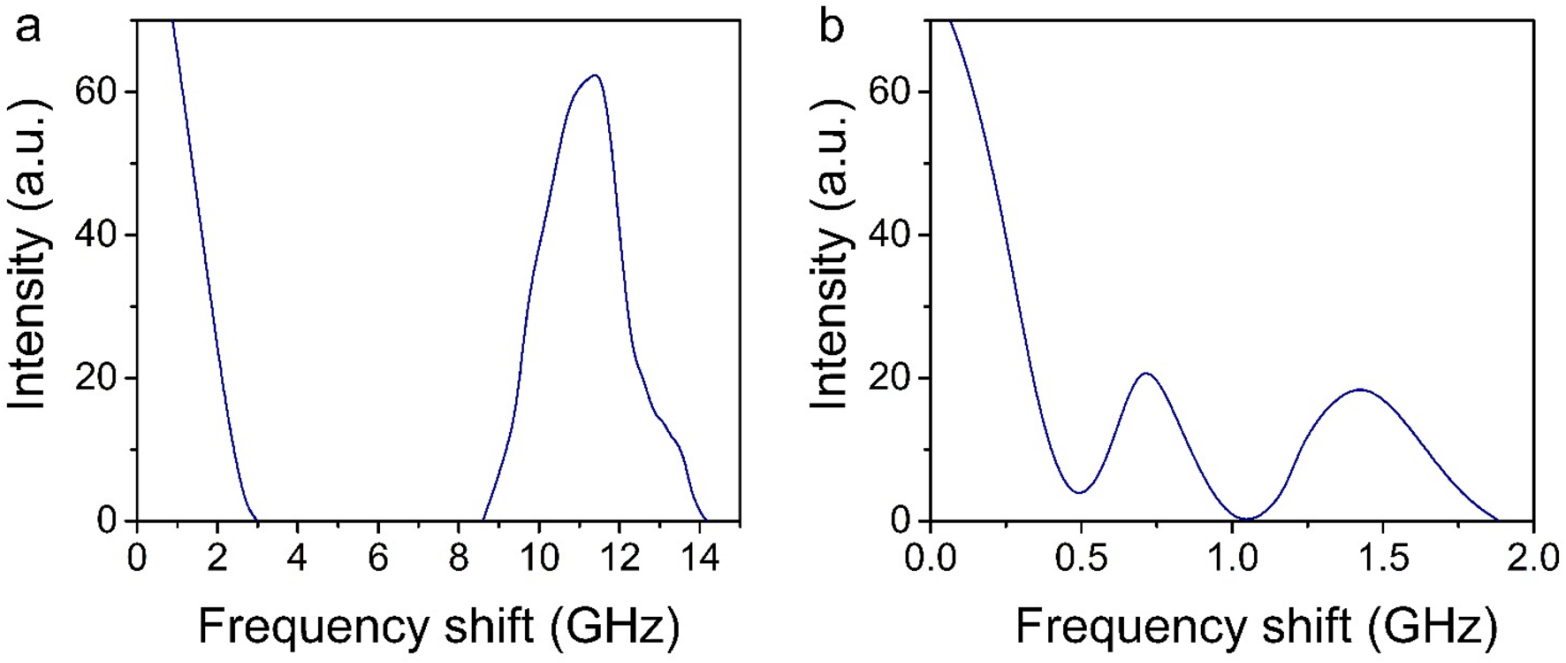
Spectrum of the forward-scattered SLFRS obtained by Fabry–Perot interferometer with the range of dispersion (**a**) 30 GHz (**b**) 5 GHz.

While the energy of the laser pulse exceeded the certain threshold value (0.01 GW/cm^2^) SLFRS was excited. Forward and backward scattered first Stokes component with frequency shift about 11.5 GHz corresponding to the quadrupolar mode of the silica sphere was registered. Maximum conversion efficiency of laser radiation into a scattered wave was 43%. When the intensity of the laser pulse reaches a value of 0.03 GW/cm^2^, lower-frequency components of 0.75 and 1.5 GHz appear in the spectrum of the scattered radiation both in forward and backward directions (Fig. 2).

So, the acoustic spectrum of the sample under investigation which was measured by SLFRS consists of the following spectral lines—11.5 GHz, 0.75 GHz and 1.5 GHz. Let us compare the experimentally obtained values of the natural frequencies of the system with the calculated values. Free isotropic elastic sphere eigenfrequencies definition was realized in^9^. The solution of the equation of motion (1) gives two types of acoustic modes: a spheroidal (SPH) and a torsional (TOR).

1 $$\rho \frac{{\partial }^{2}\overrightarrow{D}}{\partial {t}^{2}}=\left(\lambda +\mu \right)\overrightarrow{\nabla }\left(\overrightarrow{\nabla } \cdot \overrightarrow{D}\right)+\mu {\nabla }^{2}\overrightarrow{D}$$

where *D* is a lattice displacement vector, μ, λ-Lame’s constants, and ρ is the mass density. The equations obtained for spheroidal modes are^10^:

2 $$\frac{{\tan}(qa)}{\mathrm{q}a}=\frac{1}{1-({{\upnu }_{\mathrm{l}}}^{2}/4{{\upnu }_{\mathrm{t}}}^{2}){\mathrm{q}}^{2}{a}^{2}} \quad (\text{l} = 0)$$

3 $$-\frac{{Q}^{2}{a}^{2}}{2}\left(2{l}^{2}-l-1-\frac{{Q}^{2}{a}^{2}}{2}\right){j}_{l}\left(qa\right){j}_{l}\left(Qa\right)+\left({l}^{3}+2{l}^{2}-l-2-{Q}^{2}{a}^{2}\right)qa{j}_{l+1}\left(qa\right){j}_{l}\left(Qa\right)+\left({l}^{3}+{l}^{2}-2l-\frac{{Q}^{2}{a}^{2}}{2}\right)Qa{j}_{l}\left(qa\right){j}_{l+1}\left(Qa\right)+\left(2-{l}^{2}-l\right)qaQa{j}_{l+1}\left(qa\right){j}_{l+1}\left(Qa\right)=0, \quad ({l}\ne 0)$$

and for torsional modes:

4 $$\frac{d}{d\left(Qa\right)}\left(\frac{{j}_{l}\left(Qa\right)}{Qa}\right)=0 \quad (\mathrm{l}\hspace{0.17em}\ge \hspace{0.17em}0)$$

where ${v}_{l},{v}_{t}$—longitudinal and transverse sound velocities, a—sphere radius, l—orbital angular momentum quantum number, ${j}_{l}$—spherical Bessel functions of the 1st kind, ${v}_{l}q={v}_{t}Q=\omega$.

The spheroidal modes occur with a change in volume, while torsional modes occur with a constant density. The eigenfrequencies values are inversely proportional to the particle radius R

5 $$\upomega =\frac{\mathrm{\xi V}}{\mathrm{R}}$$

where V is the sound velocity, and $\upxi$ is a dimensionless parameter depending on the relation between the longitudinal and transverse sound velocities.

The eigenfrequencies for both SPH and TOR modes are described by orbital angular momentum quantum number *l* and harmonic *n*. Only the breathing (*l* = 0) and quadrupolar (*l* = 2) spheroidal modes are Raman active^16^. For our sample the calculated frequencies are shown in Table 1.

**Table 1:** Eigenfrequencies of the quartz globules forming a synthetic opal matrix.

	l = 0	l = 1	l = 2	l = 3
Spheroidal	16.5 GHz	14.1 GHz	11.4 GHz	16.9 GHz
Torsional		25.1 GHz	10.9 GHz	16.8 GHz

**Figure 3:**
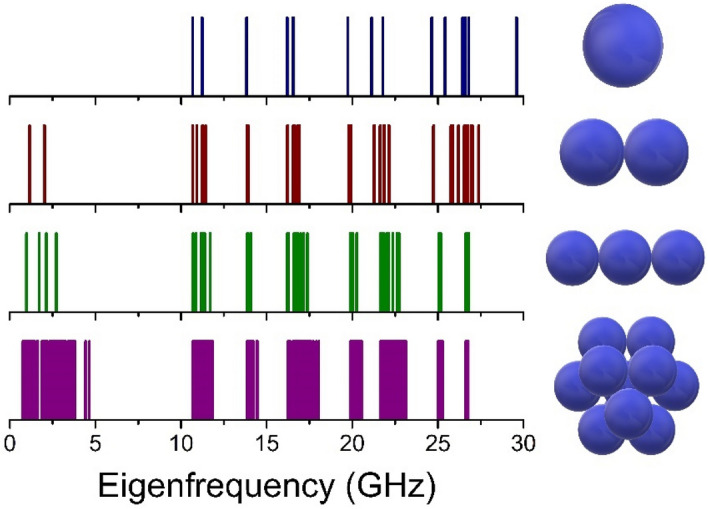
Acoustic eigenfrequencies of the system consisting of 1, 2, 3 and 12 spheres.

Point out that taking into consideration only eigen vibrations of the free quartz globule the frequencies 11.4 GHz and higher may appear in the spectra of low-frequency Raman scattering of light. Note that it would be possible to observe the torsional modes by Raman scattering if the shape of the particle was slightly asymmetric due to the presence of an odd deformation^17^. The presence of contacts existing in a real system of a synthetic opal matrix leads to the appearance of lower frequencies in the acoustic spectrum. An increase in the number of contacts leads to an increase in the number of acoustic modes in the system. Using Finite Element Method eigenfrequencies were calculated for the following cases: one free sphere, two connected spheres (1contact), sphere with two contacts and sphere with 12 contacts (as in FCC opal grid) as it is shown in Fig. 3. The radius of a sphere was 135 nm. In the case of one free sphere the lowest spectral component with frequency 11.4 GHz corresponds to the quadrupolar (l = 2) mode. When a sphere has contact with another sphere the splitting of each spectral peak is seen and new components appear in the low frequency region due to collective motions. With increasing of contacts up to 12, each peak becomes much broader and spectral bands are produced.

### Microwave generation registration

**Figure 4:**
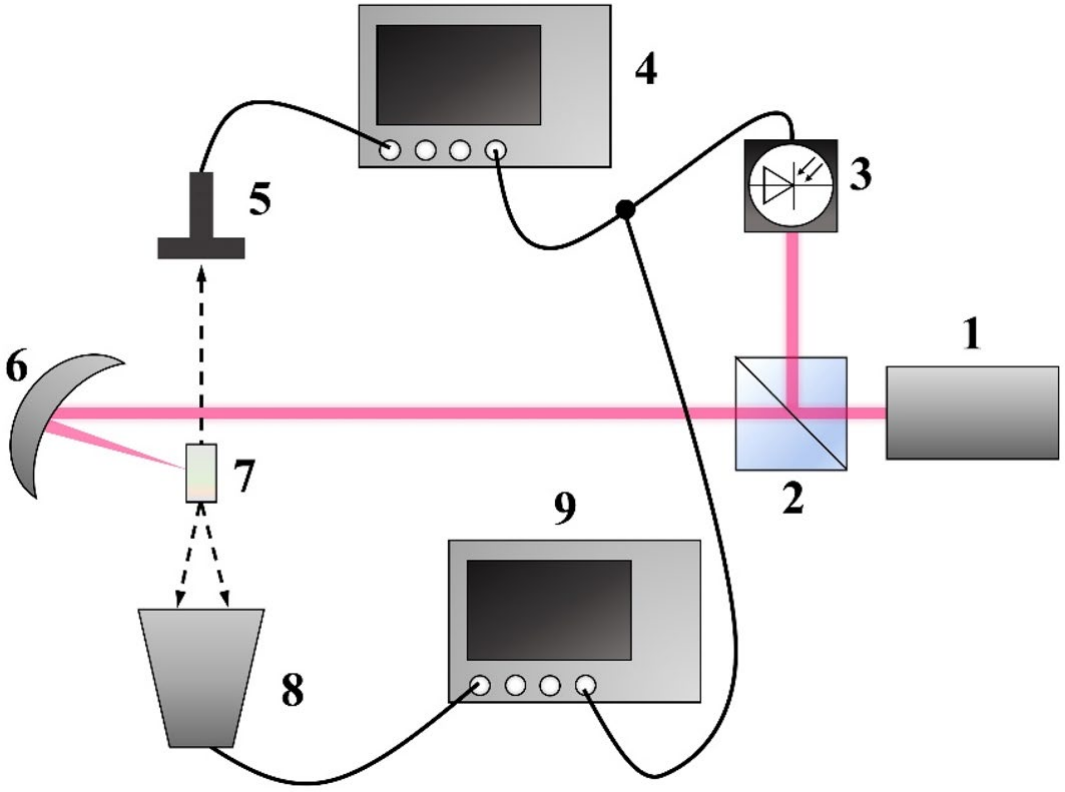
Experimental set up for microwave generation. 1—femtosecond laser, 2—beam splitter, 3—photodiode, 4—oscilloscope, 5—spiral antenna, 6—parabolic mirror, 7—opal sample, 8—wideband horn antenna, 9—spectrum analyzer.

The manifestation of the acoustic eigenfrequencies in the spectrum of SLFRS indicates the possibility obtaining stimulated generation in the microwave range by the mechanism for example specified in the work^18^. It was shown that the possibility of electromagnetic field generation at phonon frequency at the process of stimulated Raman scattering (SRS) can be realized due to nonlinear polarization modulation by optical phonons. Considering that in the SLFRS process, due to the oscillations of nano or submicron particles, their polarization is modulated, then, by analogy with the SRS process, it is possible to generate electromagnetic radiation at the frequencies of these oscillations. For submicron particles, the frequencies of these oscillations lie in the gigahertz range, which corresponds to the microwave range of the electromagnetic spectrum. For microwave radiation registration the set up presented in Fig. 4 was used.

**Figure 5:**
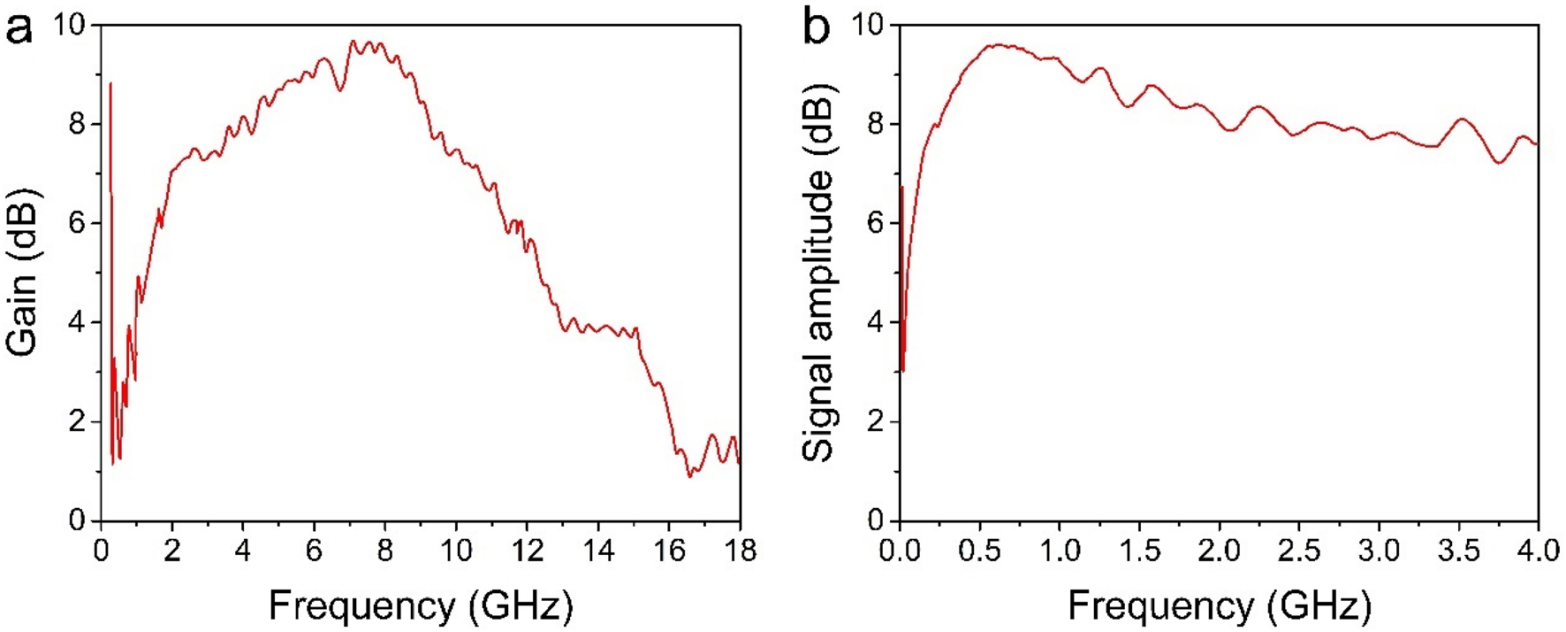
Frequency response of a horn (**a**) and a spiral (**b**) antennas.

Titanium-sapphire terawatt femtosecond laser radiation (800 nm, 35 fs laser pulse duration, peak energy 50 mJ, 10 Hz repetition rate) is focused by a parabolic mirror with focal length f = 500 mm into the opal matrix with dimensions of 5 × 5 × 4 mm. Parabolic mirror has been used because it gave possibility to obtain very sharp focusing of the exciting light on the sample (laser beam diameter in the focal plane was about 5 µm). For microwave radiation registration two types of detectors were used: a wideband horn antenna connected to a spectrum analyzer and Archimedean spiral antenna connected to oscilloscope. We used an oscilloscope with 4 GHz bandwidth, (DSO90404A) and spectrum analyzer with measurement range 9 kHz…40 GHz (8564E) equipped with low noise wideband RF-amplifier 0.3…18 GHz, 20 dB (Anritsu G3H105). Before measurements an antenna’s frequency response in this geometry was characterized (Fig. 5). In all next measurements we normalized achieved spectra to this preliminary transmission gain curve. Before an experiment we always characterized an antennas’ frequency response with calibrated RF generator in the same geometry as in our experiments. Any background noises or frequencies never were registered. When in the experiments we rotated antenna and shifted its axis from the line between sample and antenna’s axis the signal from the sample disappeared. To be sure that the signal registered with antennas is emitted by the sample we put a metal foil between the sample and antenna. In this case there was no signal.

Laser beam was partially splitted by beam splitter and routed to p-i-n photodiode for external triggering an oscilloscope and spectrum analyzer.

**Figure 6:**
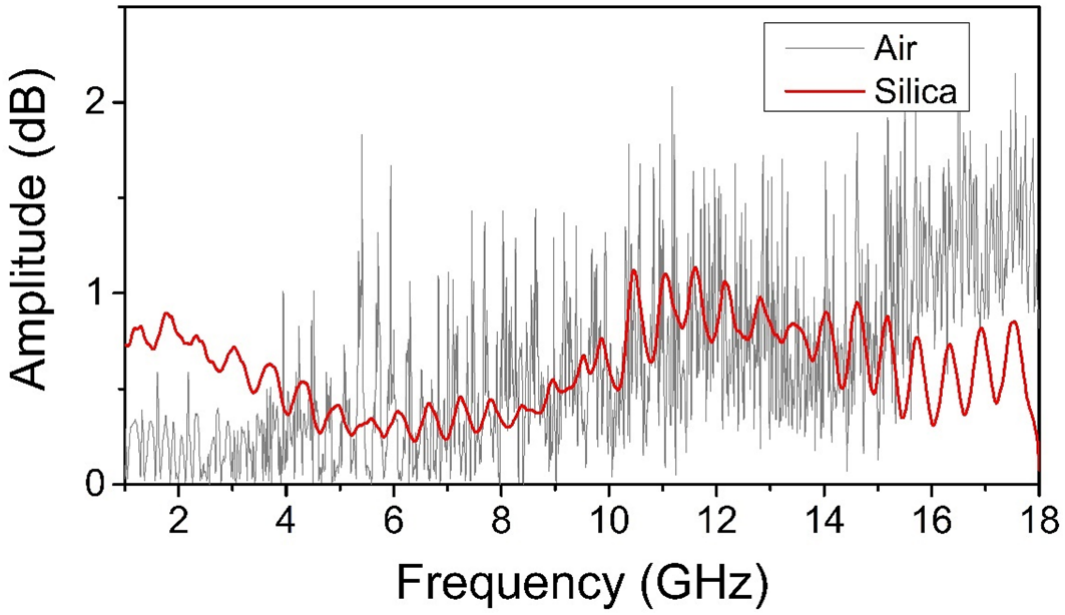
Typical EM emission power spectra registered by a wideband horn antenna in the case of laser breakdown in air and quartz glass.

When using a quartz glass sample of the same size instead of a synthetic opal matrix, the signal in the microwave range registered by a horn antenna had the form shown in Fig. 6.

**Figure 7:**
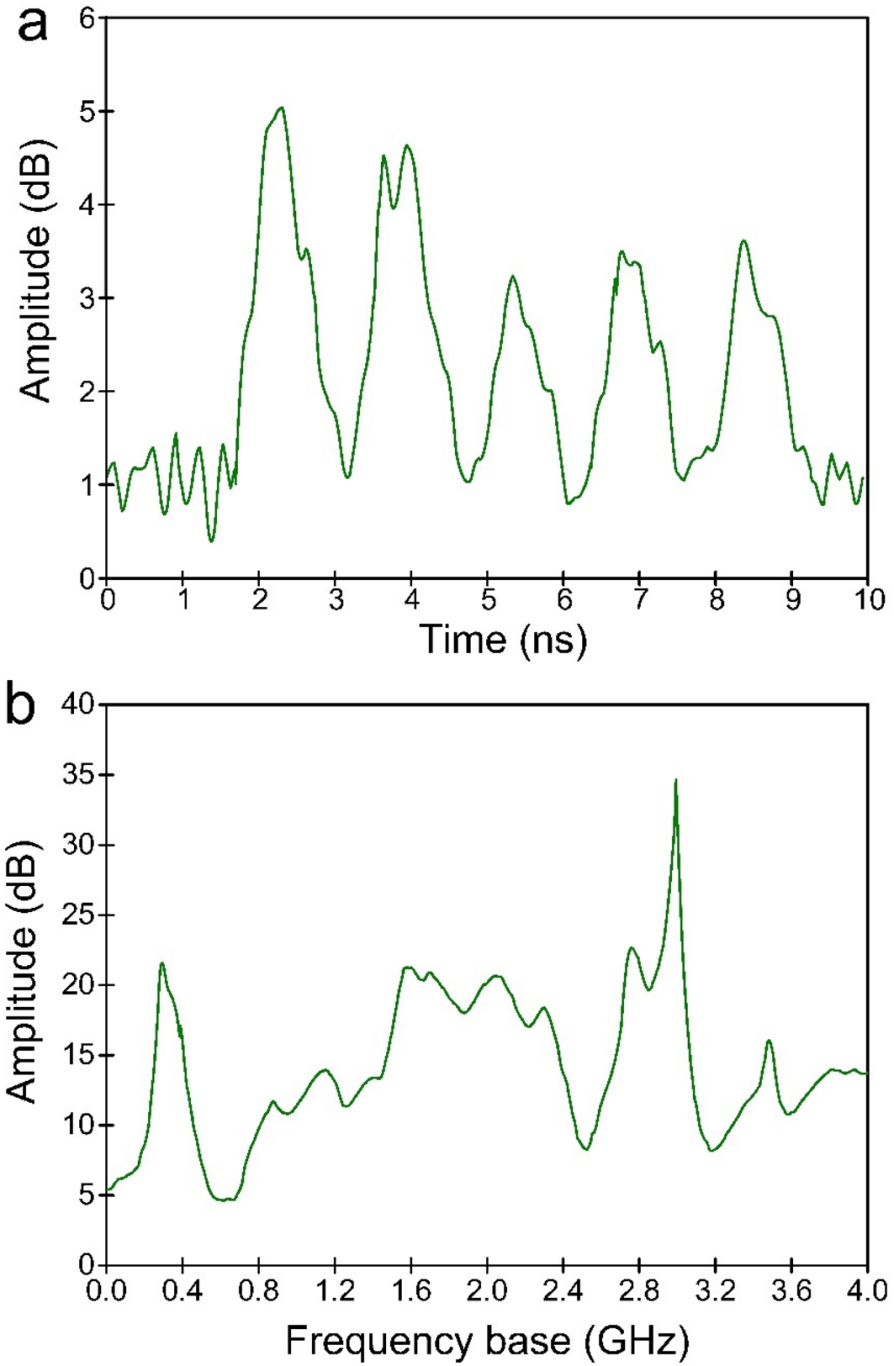
Results of the microwave emission study in opal matrix arising under laser impact; (**a**) typical oscillogram from spiral antenna; (**b**) power spectrum from spiral antenna.

In the case of using a synthetic opal sample, the radiation intensity in gigahertz range increased by about an order of magnitude. The form of the spectrum also changed. Some typical waveforms recorded in our experiments obtained using a spiral antenna and the calculated power spectrum using the Fourier transform up to 4 gigahertz are shown in Fig. 7.

**Figure 8:**
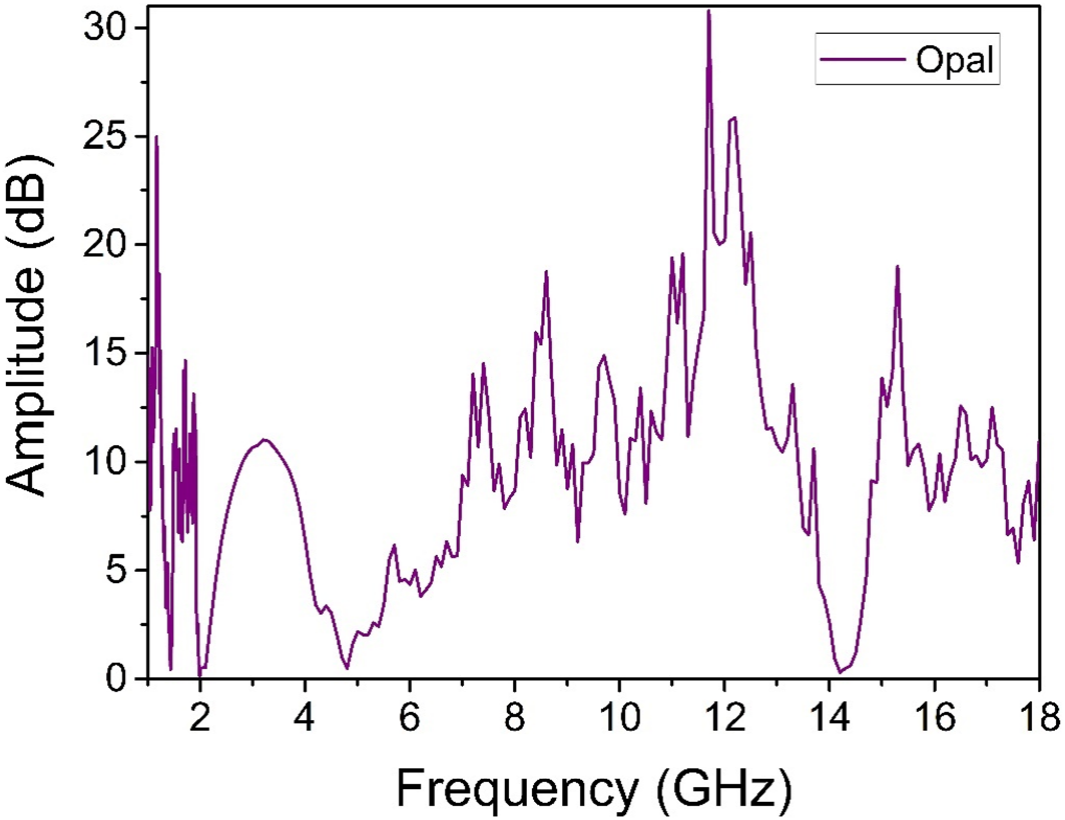
Typical EM emission power spectra registered by a wideband horn antenna.

Note the presence of several peaks in the region from 0.4 till 3 GHz which can be assigned to the acoustic frequencies obtained because of the calculation, considering the presence of contacts in the opal matrix. Typical power spectrum registered by wideband horn antenna in experiments is shown in Fig. 8. It can be seen that several peaks appear in the emission spectrum, the most intense ones which coincide with SLFRS measurements are 0.75, 1.5, and 11.5 GHz.

## Discussion

Studies of the radiation of charged or being in external electric field submicron or nanoscale particles oscillating at its own acoustic frequency are of great interest both from the point of view of understanding the physics of such fundamental and beautiful physical effects as ball lightning and St. Elmo's lights^19^, as well as for practical use in radar sensing systems. The presence of electromagnetic radiation from thunderclouds^20^ can be associated with the oscillations of charged submicron water droplets, the oscillation frequencies of which are determined by their morphology^21^. The frequency spectrum and radiation intensity of such systems are determined primarily by the size distribution of droplets. Note that any system of submicron or nanoscale particles with their own (or induced) dipole moment can emit at their own vibrational frequencies that satisfy the corresponding selection rules. It is quite difficult to separate the contribution to the radiation process in the microwave range of an oscillating submicron particle with an induced dipole moment from the contribution to the radiation of a particle of the same morphology but with a charge. It is obvious that significantly improve the efficiency of such radiation is possible in the case of the phasing of the oscillations of the particles constituting the investigated system. In the case of molecular systems such phasing is possible by stimulated Raman scattering. In the case of submicron particles while using nanosecond laser pulses the phasing of their acoustic vibrations in gigahertz range can be realized in the process of SLFRS via ponderomotive interaction^22^. Note that effective direct excitation of intrinsic acoustic vibrations of submicron particles while excited by femtosecond laser pulses may occur by the mechanism of impulsive stimulated Raman scattering (ISRS)^23–25^. For efficient generation of electromagnetic radiation by a system of submicron particles, systems with close packing and monodisperse in size are most suitable. Synthetic opal matrices (or systems with an opal structure) make it possible to obtain the maximum density of monodisperse particles in size in the range from 200 to 400 nm. Intense acoustic vibrations of quartz globules can lead to the breaking of siloxane bonds that were formed during the sintering operation of samples during their hardening. Breaking the bonds in turn leads to the formation of a charge on the surface of quartz globules. The other way for charge formation on the globules is the well-known effect of charge formation on contacting quartz surfaces^26^. Thus, the result of the interaction of pulsed laser radiation with a synthetic opal matrix is the formation of a system of charged submicron particles oscillating at the frequencies of their own acoustic vibrations. Such a system is a source of electromagnetic radiation at frequencies corresponding to its eigen acoustic frequencies.

## Conclusion

In conclusion, it should be noted that for the first time microwave radiation was generated in synthetic opal matrix via optical pumping. Several natural acoustic frequencies of globules forming synthetic opal coincide with the frequencies of the registered microwave signal. Acoustic frequencies of the sample used were measured experimentally by SLFRS and their values were verified by numerical calculations. In our opinion, the method of obtaining the generation of microwave radiation by optical pumping in systems of submicron and nanoscale particles is very promising for the creation of microwave and terahertz sources. The specific spectral range of the electromagnetic radiation generation is determined by the value of the natural acoustic frequencies of the used nano- and submicron particles, which lie in the range from gigahertz to terahertz, respectively.
